# Circulating non-coding RNA cluster predicted the tumorigenesis and development of colorectal carcinoma

**DOI:** 10.18632/aging.104055

**Published:** 2020-11-21

**Authors:** Jie Li, Yifei Feng, Ding Heng, Ranran Chen, Yong Wang, Ziwei Xu, Dongsheng Zhang, Chuan Zhang, Yue Zhang, Dongjian Ji, Junwei Tang, Yueming Sun

**Affiliations:** 1 Department of General Surgery, The First Affiliated Hospital of Nanjing Medical University, Nanjing, Jiangsu, PR China; 2 Department of Gastroenterology, The First Affiliated Hospital of Nanjing Medical University, Nanjing, Jiangsu, PR China

**Keywords:** fingerprint, non-coding RNA, biomarker, adenoma, plasma

## Abstract

Carcinoembryonic antigen (CEA) is the most significant plasma biomarker in colorectal cancer (CRC), which is mainly used to diagnose and monitor the recurrence of CRC. However, due to the low sensitivity of CEA, it is more recommended for postoperative surveillance rather than early diagnosis. It is necessary to find efficient biomarkers for CRC. In this study, the expression of plasma non-coding RNAs was confirmed in three independent cohorts with total 1201 participants. First, 12 non-coding RNAs were screened from 9 plasma samples by using microarray. The expression of selected non-coding RNAs was further validated by multiphase detection and risk score analysis. We found that miR-20b-5p, miR-329-3p, miR-374b-5p, miR-503-5p, XLOC_001120 and ENSG00000243766.2 were significantly elevated in CRC plasma, and the AUC in training and validation set was 0.996 and 0.954, respectively. Moreover, miR-20b-5p, miR-329-3p and miR-503-5p were found elevated in plasma from larger tumors (5 cm as the cutoff) in CRC patients, and the merged AUC in training and validation set was 0.896 and 0.881. In conclusion, a panel of 6 non-coding RNAs showed their important clinical value for the early diagnosis of CRC. Among, miR-20b-5p, miR-329-3p and miR-503-5p might be the potential markers for evaluating larger tumor size of CRC.

## INTRODUCTION

Colorectal cancer (CRC), one of the most common malignant tumors of the digestive tract, is characterized by high incidence, high mortality, and poor prognosis. The incidence and mortality of CRC ranks third and second in cancer, respectively [1, 2]. Despite advances in neoadjuvant therapy, radical surgery, postoperative chemoradiotherapy, and immunotherapy, the five-year survival rate of patients with CRC remains disappointed due to inefficient early diagnosis and distant metastasis [3–8]. CEA is the most significant plasma biomarker that is used to diagnose and monitor the recurrence of CRC patients. However, previous studies have investigated that the sensitivity of CEA was about 40% in clinical CRC diagnosis [9–11]. It is urgent to find an effective tool with high sensitivity and specificity for early diagnosis of patients with CRC, which can improve the prognosis of CRC.

The long non-coding RNAs (lncRNAs) and microRNAs have been extensively investigated after they were linked to initiation and progression of tumor [12–15]. Numerous studies have indicated that there are remarkable differences in the expression profiles of lncRNAs and microRNAs between the CRC tissues and normal tissues [16–19]. Further studies presented that there were lots of stable secondary structure of lncRNAs and microRNAs in body fluids, which established a theoretical foundation for uncovering their diagnostic and prognostic function of plasma lncRNAs and microRNAs in CRC [20–22]. Recent studies reported that the expression of various non-coding RNAs as diagnostic biomarkers in the plasma of CRC patients, colorectal adenoma (CRA) patients and healthy people, such as SNHG11, miR-221, miR-320d, miR-1290, miR-532-3p, miR-331, miR-195, miR-17, miR-142-3p, miR-15b, miR-532, and miR-652 [23–25]. Nevertheless, these studies were commonly restricted by one or more factors: limited number of lncRNAs or microRNAs screened, failure to distinguish CRC from CRA, without combination lncRNAs and microRNAs, and/or lack of independent large sample validation.

In this study, we aimed to investigate the circulating lncRNAs and microRNAs as biomarkers of CRC. The plasma expression profiles of lncRNAs and microRNAs were characterized by using lncRNAs and microRNAs microarray in CRC patients compare with healthy control and CRA, qRT-PCR was used to validate the differential expression of lncRNAs and microRNAs with an independent cohort of 1201 participants (597 CRC *v* 585 HC, 597 CRC *v* 19 CRA). Further analysis was conducted to confirm a panel of plasma lncRNAs, microRNAs and their combination as an efficient and stable biomarker for the diagnosis of CRC.

## RESULTS

### High throughput microarray detection of plasma lncRNAs and microRNAs

In total, 597 patients diagnosed with CRC, 585 paired healthy controls, and 19 patients diagnosed with CRA were enrolled. All participants in this study was age and gender matched. For the CRC patients, the subgroup was divided according to the Differentiation grade, tumor size (with 5cm as cutoff), with or without metastasis, and tumor TNM staging. The detailed clinical information was presented in Table 1.

**Table 1:** Clinicopathological features of surgical colorectal cancer (CRC) and cancer-free control samples.

	**CRC**	**CRA**	**Control**	***P* valve**
**N**	**597**	**19**	**585**	
Age Mean (SE) year	62.89(0.02)	57.32(0.63)	57.17(0.02)	0.32^a^
Sex (male/female)	376/221	10/9	357/228	0.55^b^
**Differentiation grade**				
Well	0			
Moderate	373			
Poorly	224			
**Tumor Size(cm)**				
≤5 cm	427			
>5 cm	170			
**Metastasis**				
Yes	288			
No	309			
**Tumor stage**				
Stage I, II	309			
Stage III, IV	288			
**TNM staging system**				
T1+T2	156			
T3+T4	441			

^a^ Student t-test.

^b^ Chi-square test.

**Figure 1:**
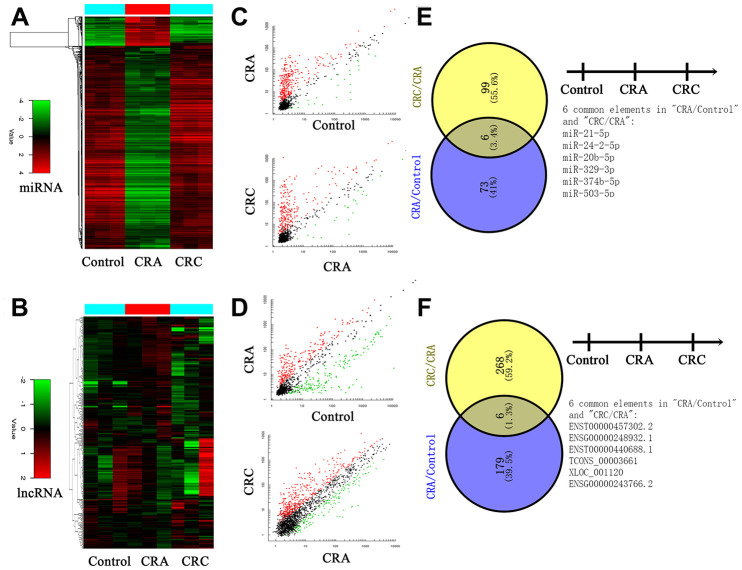
**Circulating non-coding RNA expression landscape of in HC, CRA and CRC patients.** (**A**, **B**) Cluster analysis for the miRNA and lncRNA expression in HC, CRA and CRC groups. Each group including three samples. (**C**, **D**) The scatter distribution of aberrant expressed miRNA/lncRNA in different groups. (**E**, **F**) The candidate miRNA/lncRNA was screened through Venny analysis.

First, plasma RNA was extracted from CRC group, CRA group and Control group. Samples were applied to the miRNA and lncRNA microarray. Each group we enrolled three samples. Hierarchical clustering analysis and volcano plot distribution were used to sort the aberrantly expressed miRNAs/lncRNAs in different groups. As presented in Figure 1A and 1B, different expression level of miRNA and lncRNA in each group were obtained. Then further screening was performed as follows: a, P value <0.05; b, CT value <35; c, detection rate >75%. Total of 79 miRNA transcripts were specifically increased in CRA group comparing with NC group, 105 miRNAs were collected in CRC group by comparing with CRA group. In order to screen the biomarker for predicating, the Venny analysis was applied and finally yielded 6 miRNAs candidates as listed in Figure 1C and 1E. For lncRNA, total of 185 lncRNA transcripts were specifically increased in CRA group comparing with NC group, 274 lncRNAs were collected in CRC group by comparing with CRA group. The Venny analysis finally yielded 6 lncRNAs candidates as listed in Figure 1D and 1F.

**Figure 2:**
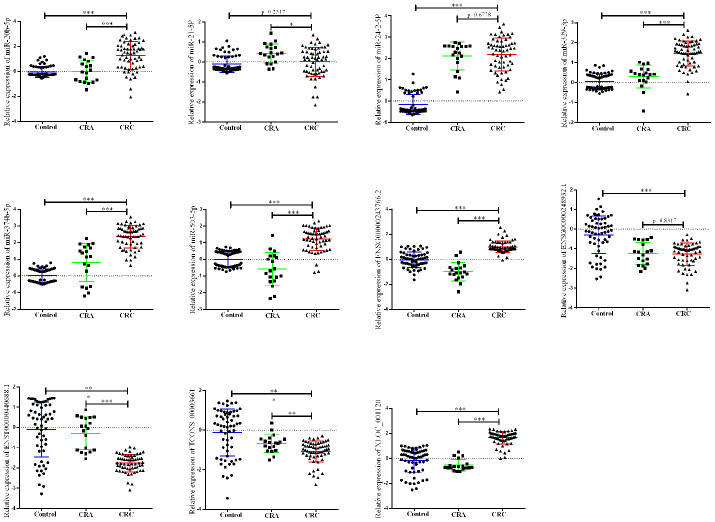
**Relative expression of candidate non-coding RNA through first-phage validation.** qRT-PCR analysis was used to detect the expression of 6 miRAN and 6 lncRNA in 40 paired plasma samples from healthy controls, 19 samples of CRA patients and 40 plasma samples from CRC patients. Data was log-transformed and was presented as mean ± SD. Data was analyzed with student t test. “***” indicated *p* < 0.001.

Next, a larger sample scale was employed for further validation the 12 candidates. As presented in Figure 2, among the 12 miRNA/lncRNA, one of which entitled with ENST00000457302.2 presented no amplification with the RT-PCR assay. Two miRNAs including miR-21-5p and miR-24-2-5p, three lncRNA including ENSG00000248932.1, ENST00000440688.1 and TCONS_00003661 presented no difference. Therefore, a panel of 6 non-coding RNAs including miR-20b-5p, miR-329-3p, miR-374b-5p, miR-503-5p, XLOC_001120 and ENSG00000243766.2 were selected the further validation analysis.

### Training set and validation set for selecting the biomarker for CRC diagnosis

**Figure 3:**
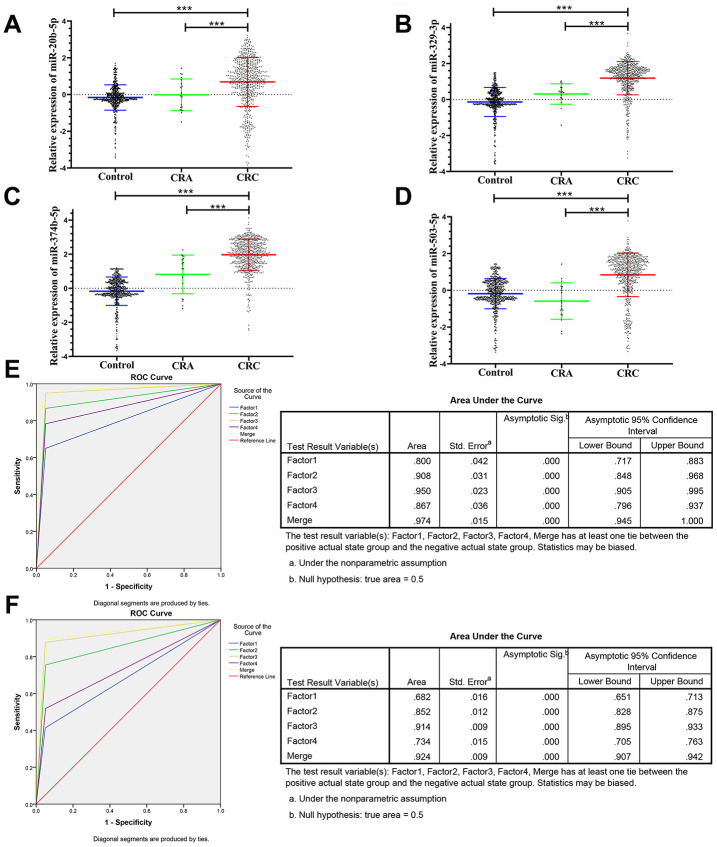
**Relative expression of 4 microRNAs in HC, CRA and CRC, and ROC curve analysis for predicting the 4 microRNAs as CRC diagnosis biomarkers.** (**A**–**D**) qRT-PCR analysis was used to detect the expression of miR-20b-5p, miR-329-3p, miR-374b-5p and miR-503-5p in 585 plasma samples from healthy controls, 19 samples of CRA patients and 597 plasma samples from CRC patients. Data was log-transformed and was presented as mean ± SD. Data was analyzed with student t test. “***” indicated *p* < 0.001. (**E**) ROC curve for the 4-microRNA signature to separate 60 CRC cases from 60 controls in the training set with the AUC presented in the right. (**F**) ROC curve analysis was used for the 4-microRNA signature to differentiate 597 CRC cases from 585 controls in the validation set with the AUC presented in the right. Factor1, 2, 3, 4 and merged represented the miR-20b-5p, miR-329-3p, miR-374b-5p, miR-503-5p and the combination of the 4 microRNAs.

**Figure 4:**
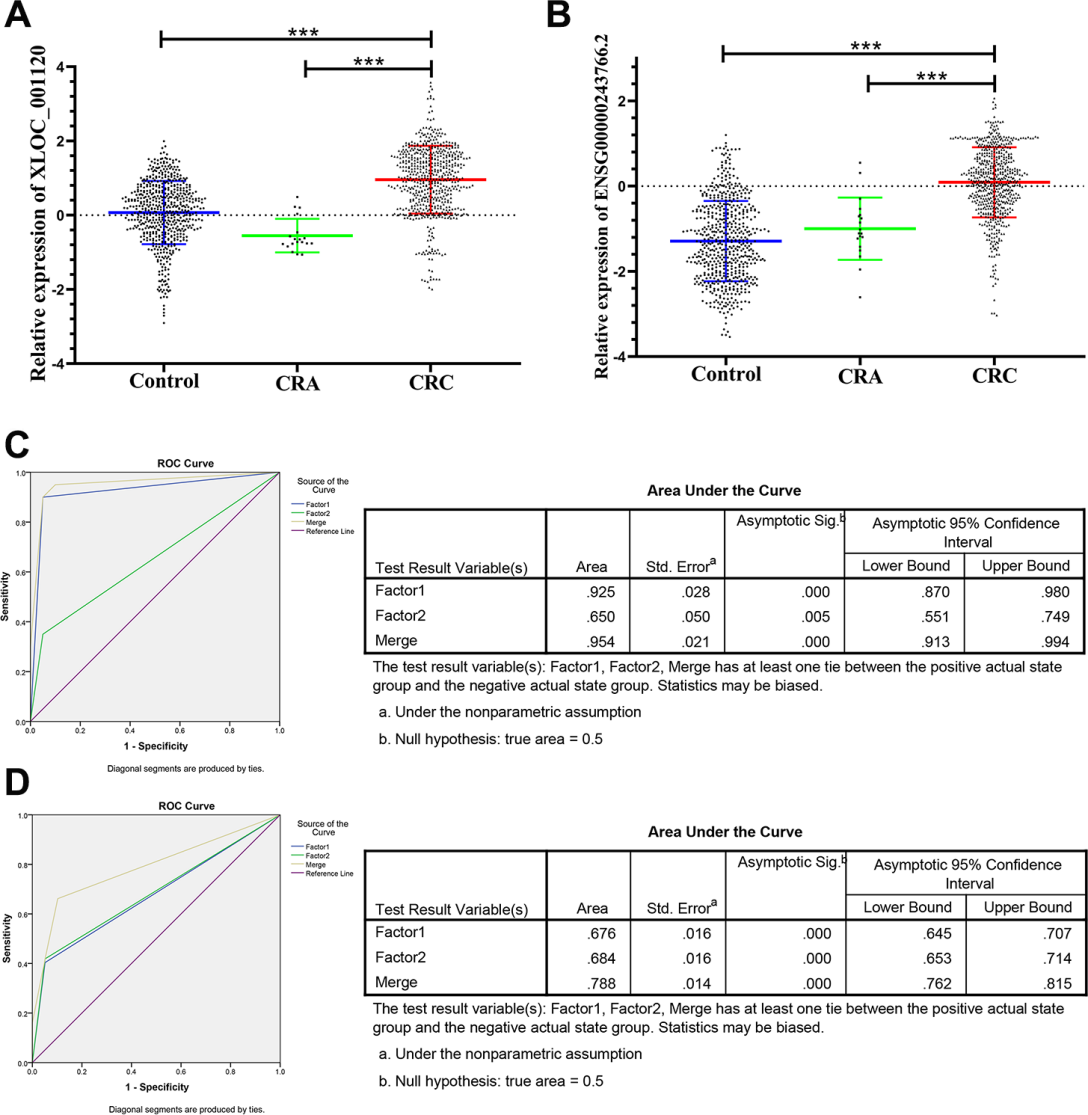
**Relative expression of 2 lncRNAs in HC, CRA and CRC, and ROC curve analysis for predicting the 2 lncRNAs as CRC diagnosis biomarkers.** (**A**–**B**) qRT-PCR analysis was used to detect the expression of XLOC_001120 and ENSG00000243766.2 in 585 plasma samples from healthy controls, 19 samples of CRA patients and 597 plasma samples from CRC patients. Data was log-transformed and was presented as mean ± SD. Data was analyzed with student t test. “***” indicated *p* < 0.001. (**C**) ROC curve for the 2-lncRNA signature to separate 60 CRC cases from 60 controls in the training set with the AUC presented in the right. (**D**) ROC curve analysis was used for the 2-lncRNA signature to differentiate 597 CRC cases from 585 controls in the validation set with the AUC presented in the right. Factor1, 2 and merged represented the XLOC_001120, ENSG00000243766.2 and the combination of the 2 lncRNAs.

The panel of 6 non-coding RNAs was found to be effective markers for the diagnosis of CRC through the abovementioned experimental design by using multiphase detection and analysis. The expression of miR-20b-5p, miR-329-3p, miR-374b-5p, miR-503-5p (Figure 3A–3D, Supplementary Tables 1 and 2) and lncRNA including XLOC_001120 and ENSG00000243766.2 were significantly increased in the CRC plasma samples compared with CRA and healthy control plasma samples (Figure 4A and 4B, Supplementary Tables 1 and 2). In addition, we also detected relative expression of miR-20b-5p, miR-329-3p, miR-374b-5p, miR-503-5p, XLOC_001120 and ENSG00000243766.2 through qRT-PCR in 60 pairs CRC tissues and matched adjacent tissues. All 6 non-coding RNAs were increased in the CRC tissues (Supplementary Figure 1A–1F).

Risk score analysis (RSA) was used to evaluate the predicting ability of the panel of 6 non-coding RNAs as CRC diagnostic markers. First, the risk score of each plasma sample were calculated and taken as a parameter for further logistic regression model. The calculated cutoff of risk score was used to divide the plasma sample into the high score group (representing predicted CRC) and the low score group (representing possible cancer-free group). Combined sensitivity and specificity were maximized at a cut-off score of 9.825, and the prediction accuracy of CRC and prediction value of cancer-free control was 0.97 and 0.97 in the training set, respectively. Then, verification of the effectiveness under the cutoff value in the larger validation samples showed the positive predictive value and negative predictive value was 0.96 and 0.77, respectively (Table 2).

**Table 2:** Risk score analysis of CRC and cancer-free control plasma samples.

**Score**	**0–9.825**	**9.825–19.65**	**PPV**	**NPV**
Training set			0.97	0.97
CRC	2	58		
Control	58	2		
Validation set			0.96	0.77
CRC	170	427		
Control	569	16		

PPV, positive predictive value.

NPV, negative predictive value.

**Figure 5:**
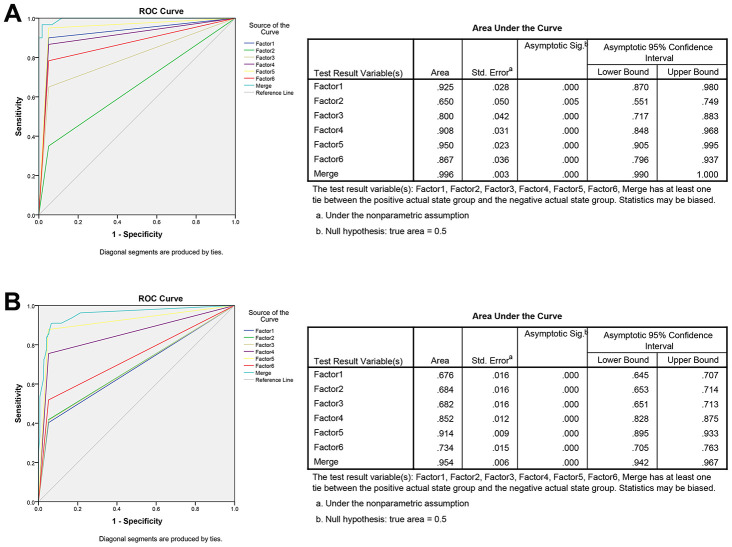
**ROC curve analysis for predicting the 6 non-coding RNAs as CRC diagnosis biomarkers.** (**A**) ROC curve for the 6 non-coding RNAs signature to separate 60 CRC cases from 60 controls in the training set with the AUC presented in the right. (**B**) ROC curve analysis was used for the 6 non-coding RNAs signature to differentiate 597 CRC cases from 585 controls in the validation set with the AUC presented in the right. Factor1, 2, 3, 4, 5, 6 and merged represented the XLOC_001120, ENSG00000243766.2, miR-20b-5p, miR-329-3p, miR-374b-5p, miR-503-5p and the combination of the 6 non-coding RNAs.

The ROC analysis was used to evaluate the diagnostic performance of the chose non-coding RNAs panel by using risk score analysis. As shown in Figures 3E, 4C and 5A, the area under the curve (AUC) of miR-20b-5p, miR-329-3p, miR-374b-5p, miR-503-5p, XLOC_001120, ENSG00000243766.2 and their combination was 0.800, 0.908, 0.950, 0.867, 0.925, 0.650 and 0.996 in training set. When the sample size expanded to 597 CRC *vs* 585 HC, the AUC for the non-coding RNAs and their combination was 0.682, 0.852, 0.914, 0.734, 0.676, 0.684 and 0.954 respectively (Figures 3F, 4D and 5B).

The panel of miR-20b-5p, miR-329-3p, miR-374b-5p, miR-503-5p, XLOC_001120 and ENSG00000243766.2 was used to differentiate the CRC and CRA by using similar risk score analysis and ROC analysis. The expression of these 6 non-coding RNAs was significantly increased in CRC plasma samples compared with the CRA plasma samples (Supplementary Table 2). The AUC of miR-20b-5p, miR-329-3p, miR-374b-5p, miR-503-5p and their combination was 0.874, 0.924, 0.861, 0.799 and 0.939 in training set, and was 0.645, 0.838, 0.713, 0.715 and 0.850 in the 597 CRC samples *vs* 19 CRA samples, respectively (Supplementary Figure 2C, 2D). As shown in Supplementary Figure 2A, 2B, The AUC of XLOC_001120, ENSG00000243766.2 and their combination was 0.749, 0.736 and 0.818 in the 40 CRC samples *vs* 19 CRA samples, and was 0.827, 0.614 and 0.869 in the validation set, respectively. A repeated validation test in the independent datasets indicated that the expression of lncRNAs and microRNAs only elevated in the plasma of CRC patients not in the CRA patients and healthy people (Supplementary Figure 5, Supplementary Table 3).

### Double-blind test for validating the diagnostic capability

80 randomly selected plasma samples (40 CRC and 40 controls) were tested in double-blind way, and classified basing on analysis of the expression of 6 non-coding RNAs in the samples by using the abovementioned diagnosis model (risk score analysis), to verify the precision of 6 non-coding RNAs as biomarkers in the diagnosis of CRC. The results showed that CRC samples were significantly separated from the control group, and the accuracy of 6 non-coding RNAs as CRC diagnostic markers was 90.0%.

### miR-20b-5p, miR-329-3p and miR-503-5p acting as the tumor size indicator via clinicopathological relevance analysis for CRC

**Figure 6:**
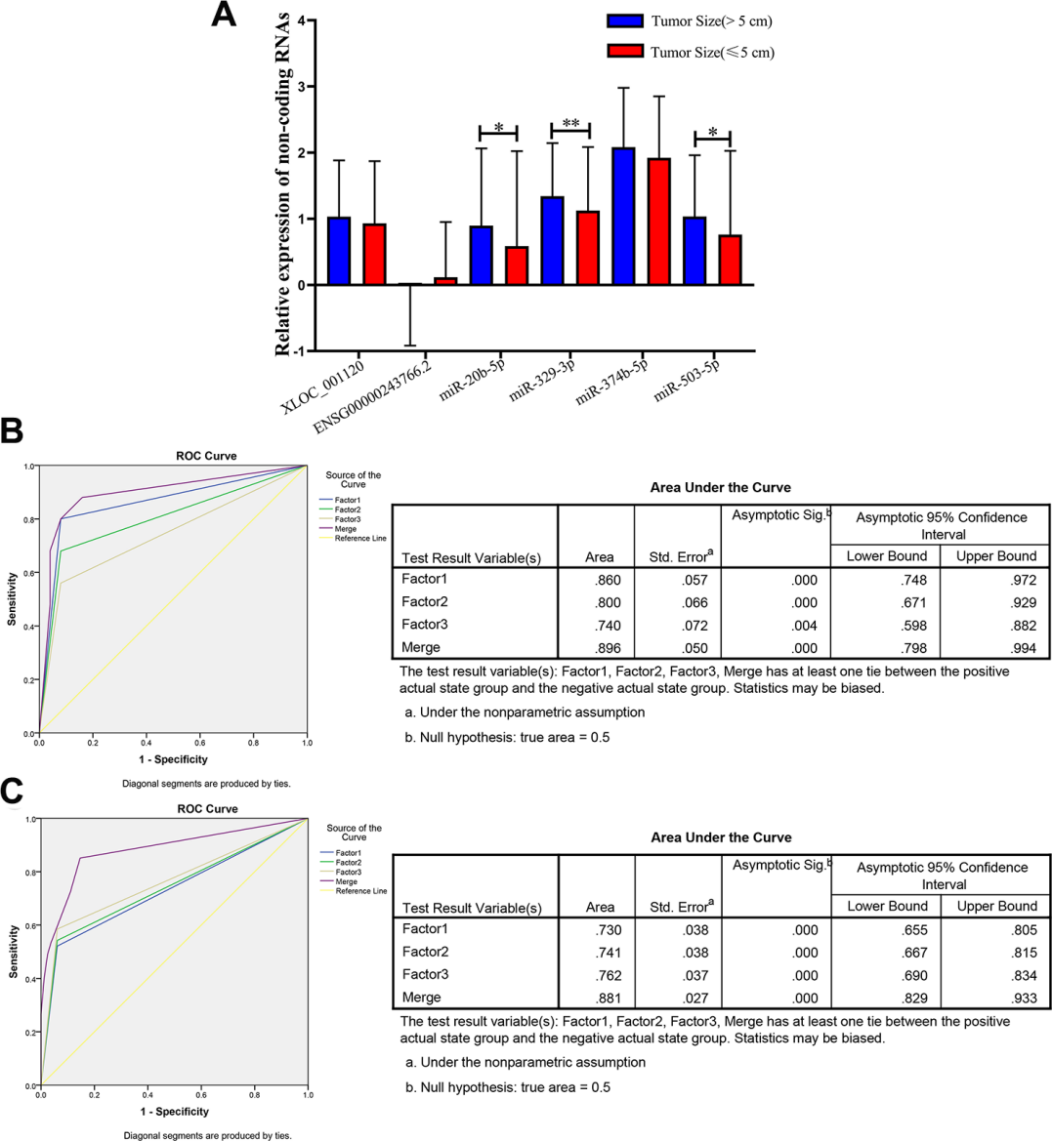
**Relative expression of 6 non-coding RNAs in different tumor size of CRC, ROC curve analysis for predicting 3 microRNAs as a CRC tumor size biomarker.** (**A**) qRT-PCR analysis was used to detect the expression of XLOC_001120, ENSG00000243766.2, miR-20b-5p, miR-329-3p, miR-374b-5p and miR-503-5p in 170 plasma samples from larger size (size>5cm) CRC patients and 427 smaller size (size≤5cm) CRC patients. Data was log-transformed and was presented as mean ± SD. Data was analyzed with student t test. “*” indicated *p* < 0.05, “**” indicated *p* < 0.01. (**B**) ROC curve analysis was conducted to discriminate between larger size group and smaller size group by the 3-microRNA profile. ROC curve analysis was performed for the 3-microRNA signature to separate 25 pairs in the training set with the AUC presented in the right. (**C**) ROC curve analysis was used for the 3-microRNA signature to differentiate 94 larger size CRC cases from 82 smaller size CRC group in the validation set with the AUC presented in the right. Factor1, 2, 3 and merged represented the miR-20b-5p, miR-329-3p, miR-503-5p and the combination of the 3 microRNAs.

Previous studies reported that the clinicopathological characteristics (including tumor size, differentiation grade, and metastasis) were significantly associated with the progression and prognosis of CRC [3, 26]. Therefore, we further analyzed the expression levels of the 6 non-coding RNAs in three following subgroups (tumor size, differentiation grade and metastasis) that based on the 597 CRC plasma samples. The results showed that there was no significant difference regarding to the tumor differentiation (well, medium or poor) and metastasis (with or without) (Supplementary Figure 3). However, 3 of the 6 non-coding RNAs, miR-20b-5p, miR-329-3p and miR-503-5p significantly elevated in plasma samples from larger tumors (5 cm as the cutoff) in CRC patients (Figure 6A).

Therefore, we randomly selected 25 (tumor size>5cm)/ 25 (tumor size≤5cm), 94 (tumor size>5cm)/ 82 (tumor size≤5cm) plasma samples as the training set and validation set of CRC to further investigate the diagnostic efficiency of miR-20b-5p, miR-329-3p and miR-503-5p. The elevated expression levels of 3 microRNAs were confirmed in the training set and validation set (Supplementary Table 4). The sensitivity and specificity of microRNAs for diagnosing larger tumor size were 94% and 75% in the training set with cutoff value 4.40, respectively. In addition, the same cutoff value was used to calculate the risk score of the validation set samples. The diagnostic sensitivity was 94%, the specificity was 64% (Table 3). The AUC of miR-20b-5p, miR-329-3p and miR-503-5p was 0.86, 0.8, 0.74 and the combination was 0.896 in training set. The AUCs in validation set were 0.73, 0.741, 0.762 and 0.881, respectively. The results indicated that the panel of three microRNAs may be a novel biomarker of diagnosis larger CRC tumor (Figure 6B and 6C).

**Table 3:** Risk score analysis of tumor size in CRC patients’ plasma samples.

**Score**	**0–4.40**	**4.40–8.80**	**PPV**	**NPV**
Training set			0.94	0.75
Size(>5 cm)	8	17		
Size(≤5 cm)	24	1		
Validation set			0.94	0.64
Size(>5 cm)	44	50		
Size(≤5 cm)	79	3		

PPV, positive predictive value.

NPV, negative predictive value.

### Stability detection of miR-20b-5p, miR-329-3p, miR-374b-5p, miR-503-5p, XLOC_001120 and ENSG00000243766.2 in human plasma

To test the stability of these 6 non-coding RNAs in human plasma, we randomly selected the 3 plasma samples from healthy controls and placed them at room temperature for 12 hours, 24 hours, and 3 cycles of fast freeze-thaw test. The results showed that all these processes had no significant effects on the concentrations of the miR-20b-5p, miR-329-3p, miR-374b-5p, miR-503-5p XLOC_001120 and ENSG00000243766.2, indicating that these non-coding RNAs were stable in human plasma (Supplementary Figure 4).

## DISCUSSION

At present, the diagnosis of CRC mainly depends on enteroscopy, imaging and tumor biomarker tests. However, the CRC patients are commonly diagnosed in the advanced stage due to the unsatisfied performance of these methods. The enteroscopy has not been able to be widely extended because of high costs, discomfort of the examination process and relatively low awareness of health in China [27]. Similarly, imaging is not suitable for large-scale screening owing to its low efficiency and high expenditures for CRC [28]. CEA has been commonly used as a plasma marker for screening and monitoring recurrence of CRC [11]. However, CEA could increase in the occurrence of intestinal inflammation, adenoma or other tumors [9, 10], leading its poor sensitivity in the detection of CRC. Therefore, it is critical to find effective tumor markers with high sensitivity and specificity for CRC diagnosis. Although previous studies have tried to find a plasma biomarker with high sensitivity and specificity, the effect of these candidates has not reached the expectation of CRC diagnosis [29]. Recent studies reported that abnormal expressed profile of lncRNAs and microRNAs in CRC tissues that pave the way for the analysis of circulating lncRNAs and microRNAs of CRC diagnosis [30, 31].

Our study discovered that plasma miR-20b-5p, miR-329-3p, miR-374b-5p, miR-503-5p, and lncRNA XLOC_001120 and ENSG00000243766.2 were efficient and stable plasma markers for diagnosing CRC by screening of the high throughput lncRNA and microRNA microarray. The non-coding RNAs panel with two lncRNAs and four microRNAs from the logistic multiple regression model, including risk score analysis and a multistage validation, represented high sensitiveness and accuracy in the diagnosis of CRC. However, the adenoma-carcinoma sequence model has been used as an essential consensus to comprehend the pathogenesis of CRC [32, 33]. In order to distinguish the differential expression profile of lncRNA and microRNA from CRC patients, we simultaneously detected the level of lncRNAs and microRNAs expression in the plasma of CRA patients. Our results and a repeated validation test in the independent datasets indicated that the expression of lncRNAs and microRNAs only elevated in the plasma of CRC patients not in the CRA patients. Then, further correlation analysis between the clinical characteristics (tumor size, differentiation grade and metastasis) and the level of these lncRNAs and microRNAs expression of CRC were performed. miR-20b-5p, miR-329-3p and miR-503-5p were recognized to be potential markers for the diagnosis of CRC tumor size by using abovementioned model. Slattery et al. demonstrated that the expression of miR-20b-5p was significantly elevated in CRC tissues compared with paired normal mucosa and overexpressed miR-20b-5p involved with NF-κB signaling pathway and apoptosis pathway [34, 35]. Previous study reported that patients with larger size tumor (≥ 6 cm) had higher levels of miR-503-5p in colon cancer [36]. Further functional study is needed to confirm the role of miR-329-3p, miR-374b-5p, XLOC_001120 and ENSG00000243766.2 in CRC.

In conclusion, we discovered a plasma non-coding RNAs panel to distinguishes CRC from healthy and adenoma. Among, miR-20b-5p, miR-329-3p and miR-503-5p showed an extra value for tumor size prediction. These plasma non-coding RNAs panel have shown significant clinical value in the early diagnosis of CRC, which could guide a timely and effective treatment to improve long-term survival.

## MATERIALS AND METHODS

### Specimens and study design

A total of 597 preoperative blood samples were collected from patients who were diagnosed with CRC and who confirmed by pathology after operation at the Department of Colorectal Surgery, the First Affiliated Hospital of Nanjing Medical University, China, in 2014-2015. In addition, 19 blood samples were obtained from patients who were confirmed with CRA and excluded CRC diagnosis at the Department of Gastroenterology during the same period. 585 blood samples from people with healthy condition confirmed by routine physical examination at the First Affiliated Hospital of Nanjing Medical University were obtained.

The written informed consent for the collection of blood specimens were obtained from each participant or their relatives. All experiments were approved by the ethic committee of the First Affiliated Hospital of Nanjing Medical University. The study was conducted in accordance with the guidelines of the Declaration of Helsinki.

High throughput microarray of lncRNAs and microRNAs in plasma samples were detected from 3 CRC patients, 3 CRA and 3 healthy controls to determine the level of lncRNAs and microRNAs in the plasma of CRC patients compared with the CRA and healthy control group. Subsequently, preliminary verification of the lncRNAs and microRNAs was performed in 60 randomly selected CRC patients and 60 healthy control plasma samples, and the candidate non-coding RNA that satisfied the conditions (P<0.05; CT value <35 and detection rate of >75%) will be further investigated. Results of these 60 pairs plasma samples were used as the training set by using the risk score analysis to calculate the relative weight of each index. The sensitivity and specificity of the candidates and their combination were calculated by receiver operating characteristic (ROC) curve analysis in diagnosing CRC. In the larger validation set (597 CRC *v* 585 HC), ROC curve analysis was conducted to verify whether the selected lncRNAs and microRNAs could also be used as efficient diagnostic markers by using the risk score analysis.

The same statistical analysis was used to determine that the selected lncRNAs and microRNAs were only significantly increased in CRC plasma samples compared to samples with colorectal adenoma in training set (40 CRC *v* 19 CRA) and validation set (597 CRC *v* 19 CRA), respectively.

The randomly selected 100 samples (including CRC patients and healthy controls) in a double-blind analysis were performed to confirm the positive predictive value and negative predictive value of the candidate lncRNAs and microRNAs based on their expression level as a filter.

The level expression of lncRNAs and microRNAs were detected in each clinical subgroup (tumor size; differentiation grade; metastasis) to identify whether the panel of lncRNAs and microRNAs could be used as molecular markers of relevant clinical characteristics of CRC.

### RNA isolation and qRT-PCR

5ml blood sample was taken and conserved in an EDTA anticoagulative tube. Full blood was firstly centrifuged at 1000 g for 10 minutes to obtain the preliminary plasma, and then centrifuged at 10,000 g for 15 minutes to remove the impurities. And the final recovered supernatant plasma was stored at -80 °C until RNA extraction.

Total RNA from plasma was extracted with TRIzol reagent (Invitrogen, USA) according to the manufacturer’s instructions. Total RNA quality and quantity was measured by NanoDrop, and then reverse transcribed into cDNA through PrimeScript RT reagent kit (Takara, Dalian, China). cDNA was then amplified with a SYBR® Premix Ex Taq™ kit (Takara) using a RT-PCR LC480 II System (Roche Diagnostics Ltd., Forrentrasse CH-6343 Rotkreuz, Switzerland) at reaction volumes of 10 μL. The results were calculated through relative quantification by the 2^−ΔΔCT^ method. The primers of the target lncRNA, microRNA and internal control were designed as follows (Supplementary Table 5). The specific Bulge-LoopTM miRNA qRT-PCR primer for microRNA and U6 were designed by Generay Biotech Co., Ltd (Shanghai, China). All reactions were run in triplicate.

### Statistical analysis

Statistical differences in clinical features (age and gender) were evaluated via Student’s t-test or χ^2^ test. The differences of lncRNA and microRNA expression in plasma (CRC *v* healthy control, CRC *v* Adenoma sample) were compared by the paired student-test. Risk score analysis was used to assess the efficiency of the two-plasma lncRNA and four-plasma microRNA signature for CRC diagnosis and metastasis predication as described [37]. Based on the training set, the preliminary diagnostic lncRNA and microRNA markers were selected by a logistic regression model. Frequency tables and receiver operating characteristic (ROC) curve were constructed to evaluate the prediction accuracy of being diagnosed with CRC. The diagnostic value of the chose lncRNA and microRNA panels was validated by the AUC that was used as a precision index. STATA 14.0 (TX, USA), SPSS software 22.0 (Chicago, USA) and Graphpad Prism 8.0 (CA, USA) performed the analyses based on the experimental design. P value less than 0.05 is statistically significant.

## Supplementary Material

Supplementary Figures

Supplementary Tables
