# Supplementary material for: Circulating non-coding RNA cluster predicted the tumorigenesis and development of colorectal carcinoma
Source: Aging (Albany NY). 2020 Nov 21;12(22):23047–66. doi: 10.18632/aging.104055 (PMC7746361; doi:10.18632/aging.104055)
Supplement: Supplementary Figures [file aging-12-104055-s001..pdf]

SUPPLEMENTARY FIGURES

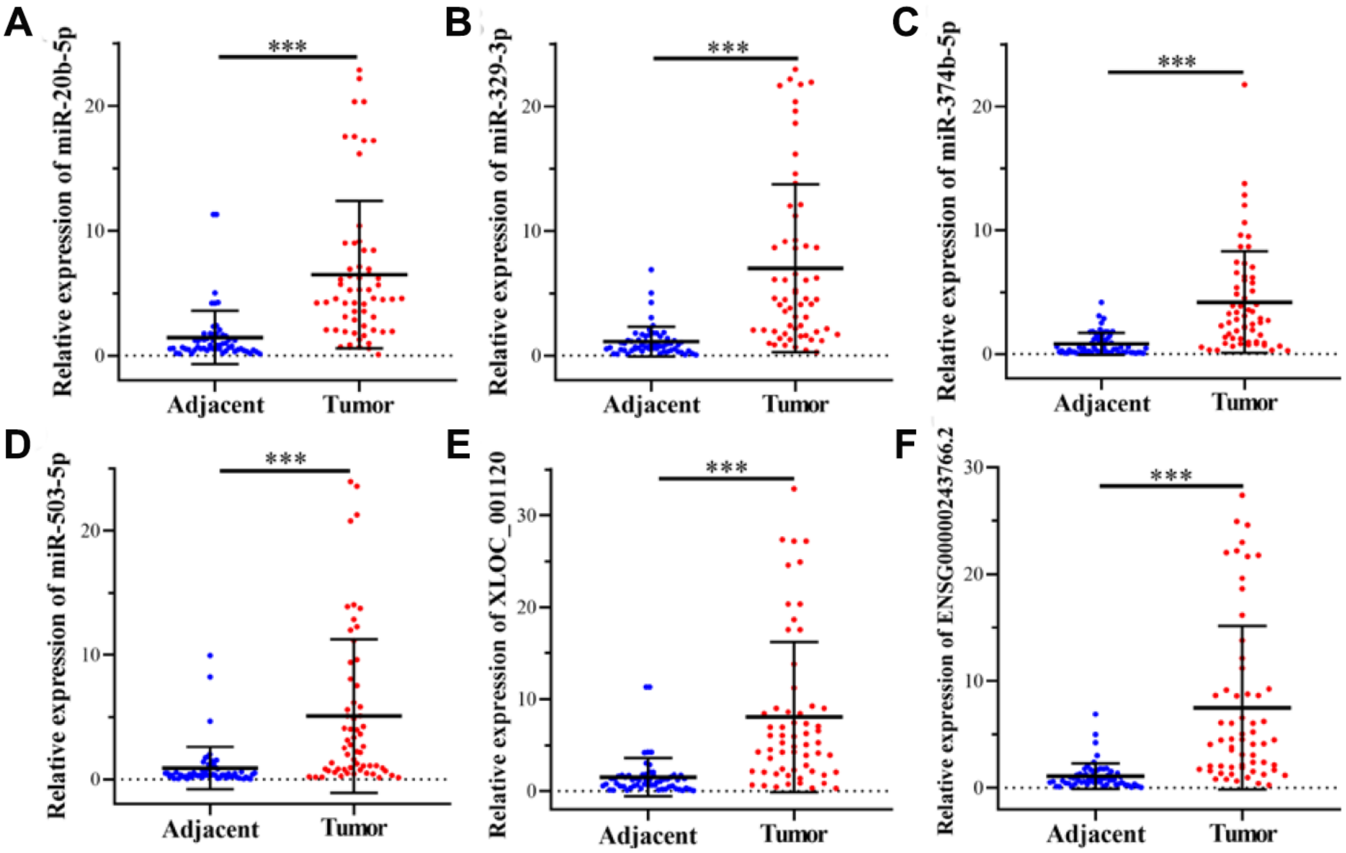

Supplementary Figure 1. Relative expression of 6 non-coding RNAs in CRC tissues and matched adjacent tissues. (**A**–**F**) The relative expression of miR-20b-5p, miR-329-3p, miR-374b-5p, miR-503-5p, XLOC_001120 and ENSG00000243766.2 were detected through qRT-PCR in 60 pairs colorectal cancer tissues and matched adjacent tissues. Data was presented as mean ± SD. Data was analyzed with student t test. ***P < 0.001.

A

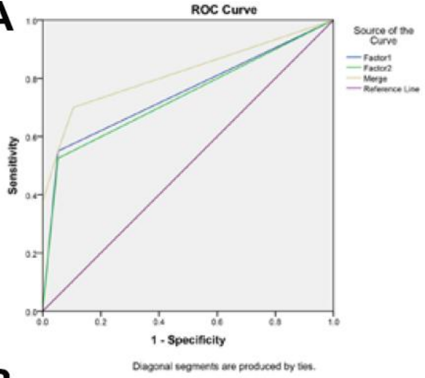

Area Under the Curve

Test Result Variable(s)	Area	Std. Error[a]	Asymptotic Sig.[b]	Asymptotic 95% Confidence Interval	
				Lower Bound	Upper Bound
Factor1	.749	.064	.002	.624	.873
Factor2	.736	.065	.004	.609	.863
Merge	.818	.054	.000	.712	.924

The test result variable(s): Factor1, Factor2, Merge has at least one tie between the positive actual state group and the negative actual state group. Statistics may be biased.

a. Under the nonparametric assumption

b. Null hypothesis: true area = 0.5

B

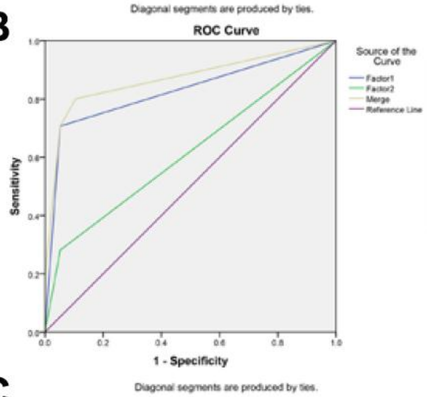

Area Under the Curve

Test Result Variable(s)	Area	Std. Error[a]	Asymptotic Sig.[b]	Asymptotic 95% Confidence Interval	
				Lower Bound	Upper Bound
Factor1	.827	.035	.000	.758	.897
Factor2	.614	.055	.089	.507	.722
Merge	.869	.030	.000	.810	.927

The test result variable(s): Factor1, Factor2, Merge has at least one tie between the positive actual state group and the negative actual state group. Statistics may be biased.

a. Under the nonparametric assumption

b. Null hypothesis: true area = 0.5

C

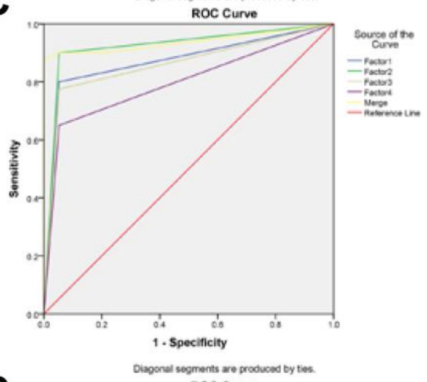

Area Under the Curve

Test Result Variable(s)	Area	Std. Error[a]	Asymptotic Sig.[b]	Asymptotic 95% Confidence Interval	
				Lower Bound	Upper Bound
Factor1	.874	.049	.000	.778	.970
Factor2	.924	.041	.000	.844	1.000
Factor3	.861	.051	.000	.762	.961
Factor4	.799	.058	.000	.684	.913
Merge	.939	.032	.000	.876	1.000

The test result variable(s): Factor1, Factor2, Factor3, Factor4, Merge has at least one tie between the positive actual state group and the negative actual state group. Statistics may be biased.

a. Under the nonparametric assumption

b. Null hypothesis: true area = 0.5

D

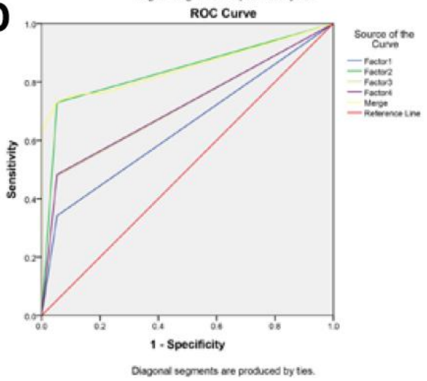

Area Under the Curve

Test Result Variable(s)	Area	Std. Error[a]	Asymptotic Sig.[b]	Asymptotic 95% Confidence Interval	
				Lower Bound	Upper Bound
Factor1	.645	.052	.032	.543	.746
Factor2	.838	.035	.000	.770	.907
Factor3	.713	.045	.002	.625	.801
Factor4	.715	.045	.001	.628	.803
Merge	.850	.023	.000	.805	.895

The test result variable(s): Factor1, Factor2, Factor3, Factor4, Merge has at least one tie between the positive actual state group and the negative actual state group. Statistics may be biased.

a. Under the nonparametric assumption

b. Null hypothesis: true area = 0.5

Supplementary Figure 2. ROC curve analysis for predicting 6 non-coding RNAs as a CRC diagnosis biomarker. (**A**) ROC curve for the 2-lncRNA signature to separate 40 CRC cases from 19 CRA cases in the training set with the AUC presented in the right. (**B**) ROC curve analysis was used for the 2-lncRNA signature to differentiate 597 CRC cases from 19 CRA cases in the validation set with the AUC presented in the right. Factor1, 2 and merged represented the XLOC_001120, ENSG00000243766.2 and the combination of the 2 lncRNAs. (**C**) ROC curve for the 4-microRNA signature to separate 40 CRC cases from 19 CRA cases in the training set with the AUC presented in the right. (**D**) ROC curve analysis was used for the 4-microRNA signature to differentiate 597 CRC cases from 19 cases in the validation set with the AUC presented in the right. Factor1, 2, 3, 4 and merged represented the miR-20b-5p, miR-329-3p, miR-374b-5p, miR-503-5p and the combination of the 4 microRNAs.

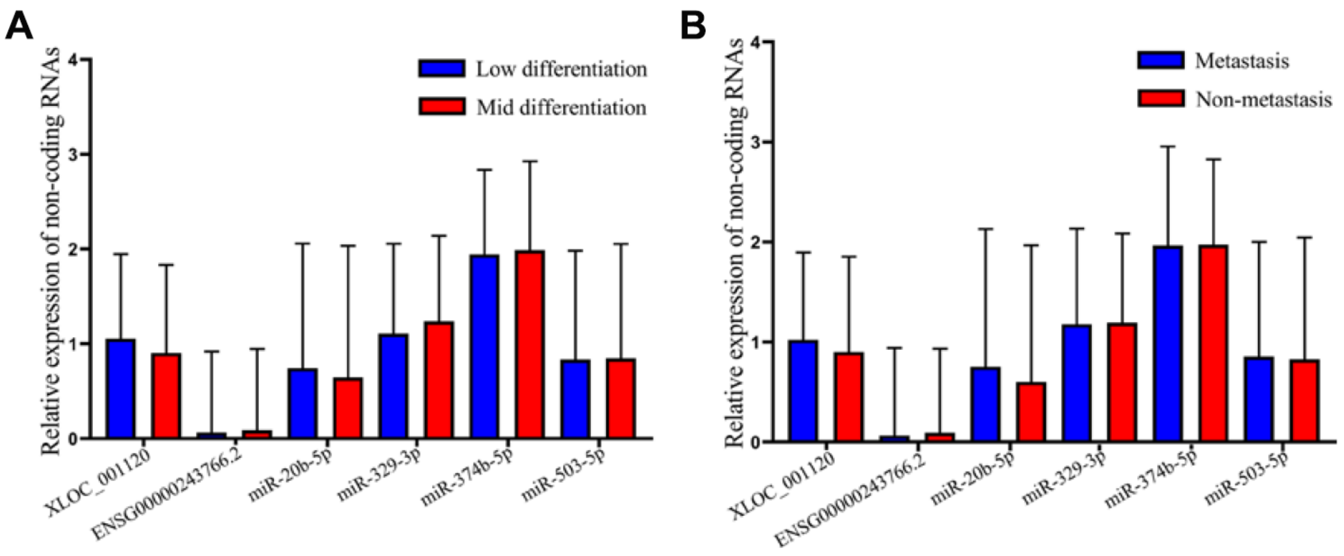

Supplementary Figure 3. Relative expression of 6 non-coding RNAs in subgroup of CRC. (**A**) CRC patients were divided by the differentiation of tumor accoding to the pathological diagnosis (373 Mid differentiation vs 224 Low differentiation). (**B**) CRC patients were separated into two groups (288 Metastasis vs 309 Non-metastasis). Data was log-transformed and was presented as mean ± SD, was analyzed with student t test. No significant difference was obtained in subgroup.

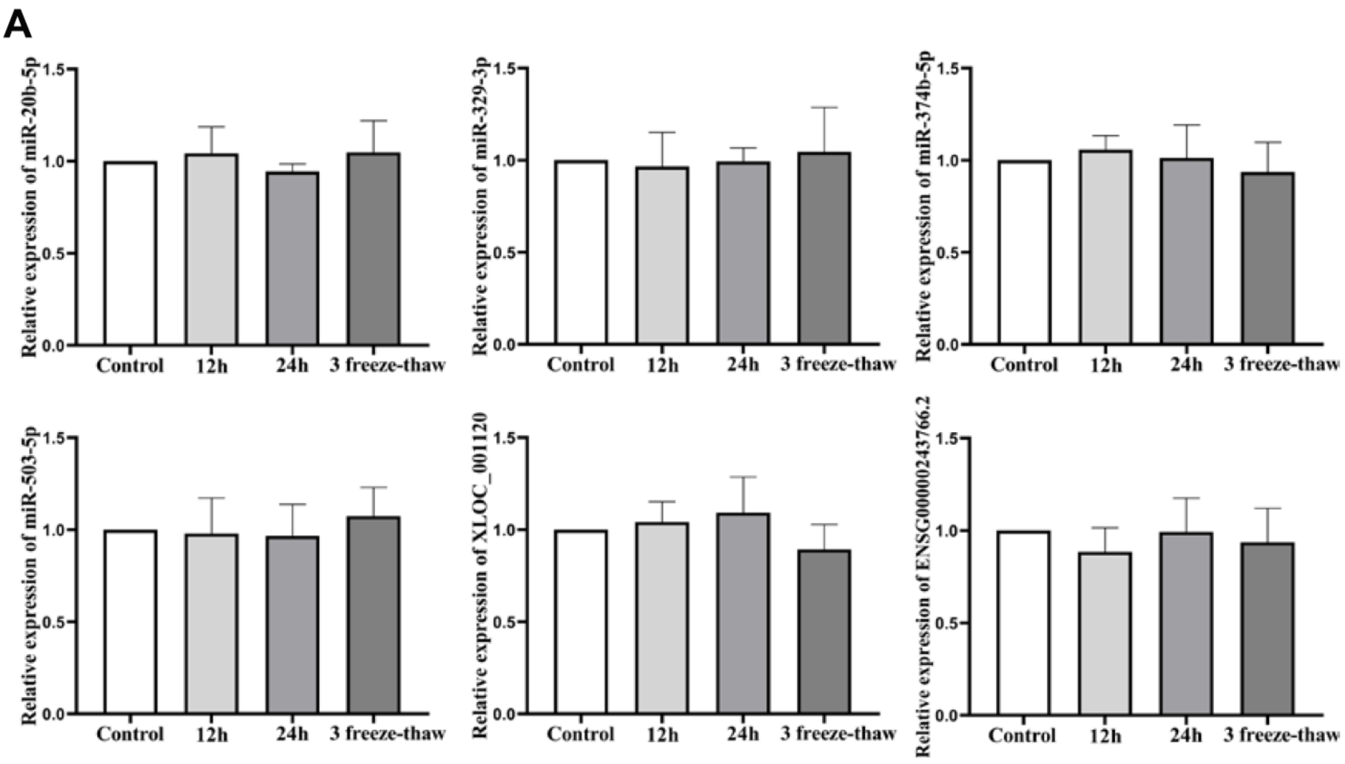

Supplementary Figure 4. Stability test of 6 non-coding RNAs in human plasma. (**A**) The expression of miR-20b-5p, miR-329-3p, miR-374b-5p, miR-503-5p, XLOC_001120 and ENSG00000243766.2 was detected under the condition that plasma samples were placed at room temperature for 12 hours, 24 hours, and 3 cycles of fast freeze-thaw by using qRT-PCR, respectively. Data was presented as mean ± SD, was analyzed with student t test. No significant difference was obtained in each group.

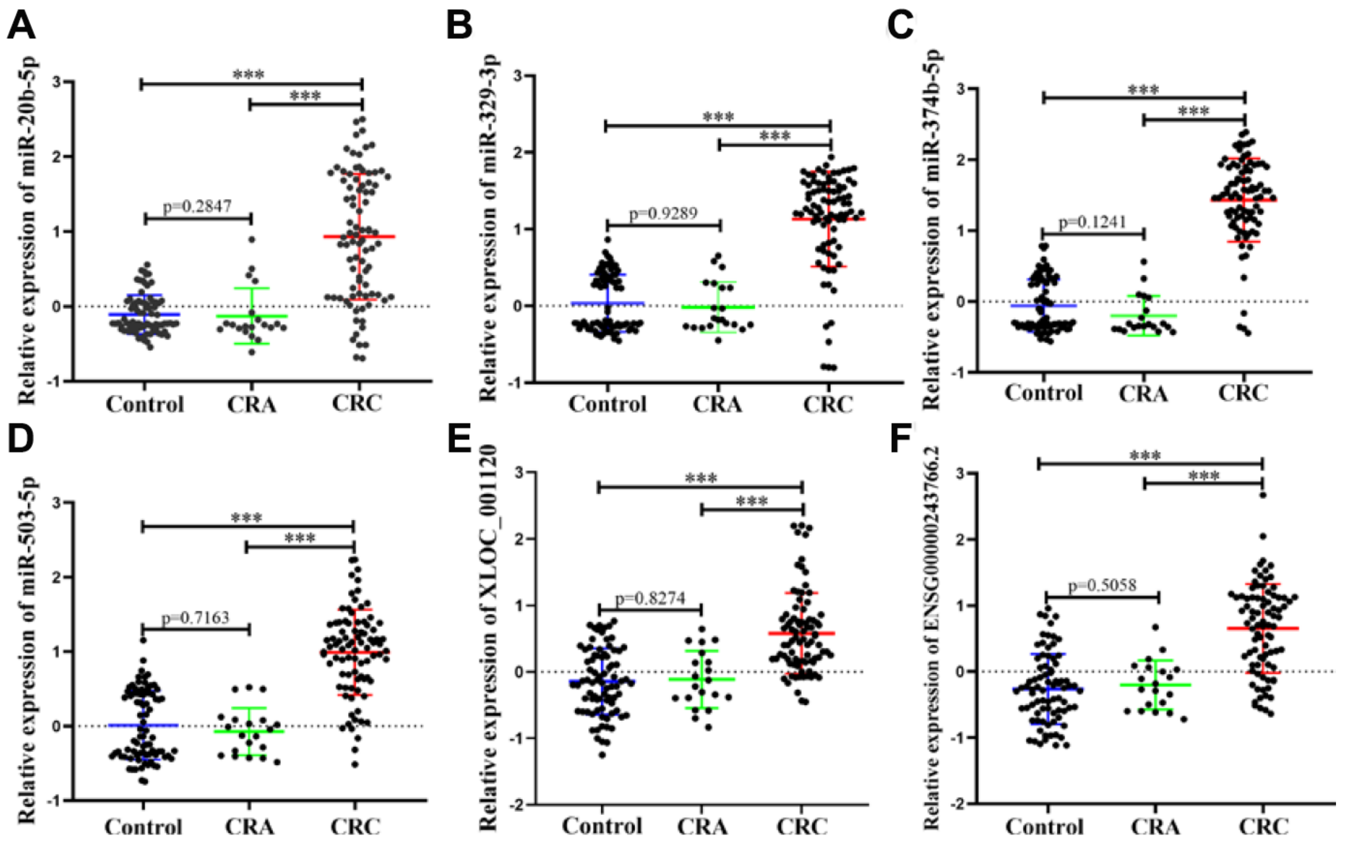

Supplementary Figure 5. Relative expression of 6 non-coding RNAs in HC, CRA and CRC in the datasets. (**A**–**F**) qRT-PCR analysis was used to detect the expression of miR-20b-5p, miR-329-3p, miR-374b-5p, miR-503-5p, XLOC_001120 and ENSG00000243766.2 in 80 plasma samples from healthy controls, 20 samples of CRA patients and 85 plasma samples from CRC patients. Data was log-transformed and was presented as mean ± SD. Data was analyzed with student t test. "***" indicated $p < 0.001$.
