# Supplementary material for: Circulating non-coding RNA cluster predicted the tumorigenesis and development of colorectal carcinoma
Source: Aging (Albany NY). 2020 Nov 21;12(22):23047–66. doi: 10.18632/aging.104055 (PMC7746361; doi:10.18632/aging.104055)
Supplement: Supplementary Tables [file aging-12-104055-s002..pdf]

SUPPLEMENTARY TABLES

Supplementary Table 1. The primers for RT-qPCR of lncRNAs.

hsa-XLOC_001120-F	GCGGGCTTAGTAGCTTCAGG
hsa-XLOC_001120-R	GTTGGGTAGTTGCCGTCTCC
hsa-ENSG00000243766.2-F	TCCCTGTGCACCATTCATCC
hsa-ENSG00000243766.2-R	CAGGTCCGGTCCACAAAGAA
hsa-ENSG00000248932.1-F	AACGAAGTGCCTAATCCCCG
hsa-ENSG00000248932.1-R	CTGGAGACTCGTTTCGCCTT
hsa-ENST00000440688.1-F	AGCCACATGGCTCAGGATTC
hsa-ENST00000440688.1-R	CGCCACTCCATAGTCACCAG
hsa-TCONS_00003661-F	GGGTGACTCACTGAAGACGG
hsa-TCONS_00003661-R	ATAATCGCACAGGCAGAGGG
hsa-ENST00000457302.2-F	TGTGACCTGAGGGACTGAAC
hsa-ENST00000457302.2-R	AAGCCATTAGCCACAGGGAAA
hsa-GAPDH-F	GGACCTGACCTGCCGTCTAG
hsa-GAPDH-R	GTAGCCCAGGATGCCCTTGA

The primers for reverse transcription PCR of microRNAs.

hsa-miR-20b-5p	CCTGTTGTCTCCAGCCACAAAAGAGCACAATATTCAGGAGACAACAGGCTACCTG
hsa-miR-21-5p	CCTGTTGTCTCCAGCCACAAAAGAGCACAATATTCAGGAGACAACAGGTCAACAT
hsa-miR-24-2-5p	CCTGTTGTCTCCAGCCACAAAAGAGCACAATATTCAGGAGACAACAGGCTGTGTT
hsa-miR-329-3p	CCTGTTGTCTCCAGCCACAAAAGAGCACAATATTCAGGAGACAACAGGAAAGAGG
hsa-miR-374b-5p	CCTGTTGTCTCCAGCCACAAAAGAGCACAATATTCAGGAGACAACAGGCACTTAG
hsa-miR-503-5p	CCTGTTGTCTCCAGCCACAAAAGAGCACAATATTCAGGAGACAACAGGCTGCAGA
Homo-U6	AACGCTTCACGAATTTGCGT

The primers for RT-qPCR of microRNAs.

hsa-miR-20b-5p-F	CGCCGCAAAGTGCTCATAGTG
hsa-miR-20b-5p-R	CAGCCACAAAAGAGCACAAT
hsa-miR-21-5p-F	CGGGCTAGCTTATCAGACTG
hsa-miR-21-5p-R	CAGCCACAAAAGAGCACAAT
hsa-miR-24-2-5p-F	CGCCGTGCCTACTGAGCTGA
hsa-miR-24-2-5p-R	CAGCCACAAAAGAGCACAAT
hsa-miR-329-3p-F	GCGGCAACACACCTGGTTAA
hsa-miR-329-3p-R	CAGCCACAAAAGAGCACAAT
hsa-miR-374b-5p-F	GCGGCATATAATACAACCTG
hsa-miR-374b-5p-R	CAGCCACAAAAGAGCACAAT
hsa-miR-503-5p-F	CGGGCTAGCAGCGGGAACAGT
hsa-miR-503-5p-R	CAGCCACAAAAGAGCACAAT
Homo-U6-F	CTCGCTTCGGCAGCACA
Homo-U6-R	AACGCTTCACGAATTTGCGT

Supplementary Table 2. Non-coding RNAs expression levels in CRC and cancer-free control plasma samples in the training and validation sets.

Non-coding RNAs	Training set			Validation set		
	CRC[a]	Control[a]	P^b	CRC[a]	Control[a]	P^b
N	60	60		597	585	
XLOC_001120	58.58 (31.61- 91.15)	1.26 (0.14-3.74)	$< 1 \times 10^{-10}$	10.63 (2.37-40.93)	1.48 (0.39-4.57)	$< 1 \times 10^{-10}$
ENSG00000243766.2	7.15 (5.69-20.79)	0.98 (0.37-2.22)	$< 1 \times 10^{-10}$	1.39 (0.41-4.89)	0.045 (0.0083-0.27)	$< 1 \times 10^{-10}$
miR-20b-5p	30.22 (2.16-112.40)	0.55 (0.48-2.08)	$< 1 \times 10^{-10}$	7.50 (1.19-44.33)	0.60 (0.43-1.29)	$< 1 \times 10^{-10}$
miR-329-3p	34.96 (10.08-84.37)	0.64 (0.51-2.52)	$< 1 \times 10^{-10}$	25.68 (7.97-54.10)	0.81 (0.47-2.24)	$< 1 \times 10^{-10}$
miR-374b-5p	266.9 (84.80-727.9)	0.57 (0.44-2.41)	$< 1 \times 10^{-10}$	120.47 (28.99-438.83)	0.78 (0.39-2.25)	$< 1 \times 10^{-10}$
miR-503-5p	21.56 (5.91-49.34)	0.56 (0.36-2.75)	$< 1 \times 10^{-10}$	14.75 (2.85-44.17)	0.66 (0.31-2.56)	$< 1 \times 10^{-10}$

[a] Data are expressed as the median (interquartile range).
[b] Wilcoxon rank sum test.

Supplementary Table 3. Non-coding RNAs expression levels of different tumor size in CRC patients' plasma samples in the training and validation sets.

Non-coding RNAs	Training set			Validation set		
	Big[a] (size>5cm)	Small[a] (size≤5cm)	P^b	Big[a] (size>5cm)	Small[a] (size≤5cm)	P^b
No.	25	25		94	82	
miR-20b-5p	53.26 (22.55-196.04)	1.85 (0.75-4.39)	2.71×10^{-8}	18.06 (7.65-53.26)	2.07 (0.78-4.39)	$< 1 \times 10^{-10}$
miR-329-3p	40.93 (24.93-88.95)	6.04 (3.17-15.73)	0.000003	39.60 (23.67-59.30)	9.92 (4.11-18.51)	$< 1 \times 10^{-10}$
miR-503-5p	17.15 (7.65-40.93)	3.00 (1.57-5.42)	0.000009	24.55 (9.16-45.57)	6.37 (3.00-9.16)	$< 1 \times 10^{-10}$

[a] Data are expressed as the median (interquartile range).
[b] Wilcoxon rank sum test.

Supplementary Table 4. Non-coding RNAs expression levels in CRC and CRA plasma samples.

Non-coding RNAs	CRC[a] (*N*=597)	CRA[a] (*N*=19)	P^b
XLOC_001120	10.63 (2.37-40.93)	0.19 (0.17-0.35)	8.22×10^{-10}
ENSG00000243766.2	1.39 (0.41-4.89)	0.078 (0.038-0.21)	2.80×10^{-7}
miR-20b-5p	7.50 (1.19-44.33)	0.84 (0.15-6.34)	0.003
miR-329-3p	25.68 (7.97-54.10)	2.60 (1.27-4.63)	2.74×10^{-7}
miR-374b-5p	120.47 (28.99-438.83)	15.03 (0.24-54.19)	0.000012
miR-503-5p	14.75 (2.85-44.17)	0.21 (0.05-1.58)	5.44×10^{-7}

[a] Data are expressed as the median (interquartile range).
[b] Wilcoxon rank sum test.

Supplementary Table 5. Clinicopathological features of surgical colorectal cancer (CRC) and cancer-free control samples in independent datasets.

N	CRC	CRA	Control	*P* valve
	**85**	**20**	**80**	
Age Mean (SE) year	60.23(0.09)	58.43(0.29)	59.17(0.12)	0.64[a]
Sex (male/female)	46/39	12/8	42/38	0.43[b]
**Differentiation grade**				
Well	0			
Moderate	42			
Poorly	43			
**Tumor Size(cm)**				
≤5 cm	51			
>5 cm	34			
**Metastasis**				
Yes	38			
No	47			
**Tumor stage**				
Stage I, II	47			
Stage III, IV	38			
TNM staging system				
T1+T2	35			
T3+T4	50			

[a] Student t-test.
[b] Chi-square test.
